# Antibiotic-Driven Gut Microbiome Dysbiosis: Resistome Dynamics, Metabolic Disruption, and Paths to Restoration

**DOI:** 10.3390/antibiotics15070688

**Published:** 2026-07-15

**Authors:** Adelina-Gabriela Niculescu, Cristina-Maria Iacob, Elvira Brătilă, Raluca Tocariu, Ciprian Andrei Coroleucă, Nicolae Corcionivoschi, Corneliu Ovidiu Vrancianu, Delia-Laura Popescu, Gabriela Loredana Popa, Mircea Ioan Popa, Roxana-Elena Cristian, Georgiana-Alexandra Grigore

**Affiliations:** 1 Research Institute of the University of Bucharest—ICUB, University of Bucharest, 050657 Bucharest, Romania; adelina.niculescu@upb.ro (A.-G.N.); roxana.cristian@incdsb.ro (R.-E.C.); grigore.georgiana-alexandra@s.bio.unibuc.ro (G.-A.G.); 2 Department of Science and Engineering of Oxide Materials and Nanomaterials, National University of Science and Technology Politehnica Bucharest, 011061 Bucharest, Romania; 3 Doctoral School, “Carol Davila” University of Medicine and Pharmacy, 050474 Bucharest, Romania; iacobcristinamaria20@gmail.com; 4 “Prof. Dr. Panait Sîrbu” Obstetrics and Gynecology Hospital, 060251 Bucharest, Romania; elvira.bratila@umfcd.ro (E.B.); raluca.tocariu@drd.umfcd.ro (R.T.); ciprian.coroleuca@umfcd.ro (C.A.C.); 5 Obstetrics and Gynecology Department, Faculty of Medicine, “Carol Davila” University of Medicine and Pharmacy, 050474 Bucharest, Romania; 6 Bacteriology Branch, Veterinary Sciences Division, Agri-Food and Biosciences Institute, Belfast BT4 3SD, UK; nicolae.corcionivoschi@afbini.gov.uk; 7 Academy of Romanian Scientists, 050044 Bucharest, Romania; 8 National Institute of Research and Development for Biological Sciences, 060031 Bucharest, Romania; 9 Department of Interdisciplinary Studies, Faculty of Interdisciplinary Studies, University of Bucharest, 010798 Bucharest, Romania; delia.popescu@unibuc.ro; 10 Department of Parasitology, “Carol Davila” University of Medicine and Pharmacy, 020021 Bucharest, Romania; gabriela.popa@umfcd.ro; 11 Parasitic Disease Department, Colentina Clinical Hospital, 020125 Bucharest, Romania; 12 Department of Microbiology II, “Carol Davila” University of Medicine and Pharmacy, 020021 Bucharest, Romania; mircea.ioan.popa@gmail.com; 13 Department of Microbiology, Cantacuzino National Military Medical Institute for Research and Development, 050096 Bucharest, Romania; 14 Department of Botany and Microbiology, Faculty of Biology, University of Bucharest, 050095 Bucharest, Romania; 15 Department of Oncologic Dermatology, “Elias” Emergency University Hospital, “Carol Davila” University of Medicine and Pharmacy, 020021 Bucharest, Romania

**Keywords:** gut microbiome, antibiotic-induced dysbiosis, resistome dynamics, antimicrobial resistance, metabolic disruption, microbiome resilience, microbiome restoration, mobile genetic elements

## Abstract

The gut microbiome is a dynamic ecosystem that plays essential roles in host metabolism, immune regulation, colonization resistance, and maintenance of intestinal homeostasis. Antibiotic exposure profoundly disrupts this ecosystem by reducing microbial diversity, depleting beneficial commensals, reshaping microbial metabolic functions, and remodeling the gut resistome through the selection and dissemination of antibiotic resistance genes (ARGs). Increasing evidence from longitudinal metagenomic, multi-omics, and experimental studies indicates that these perturbations may persist long after antibiotic withdrawal due to incomplete ecological recovery, sustained mobile genetic element-mediated ARG dissemination, and altered microbiome resilience. Beyond antimicrobial resistance, antibiotic-induced dysbiosis has been associated with reduced short-chain fatty acid production, altered bile acid metabolism, impaired epithelial barrier function, and broader disturbances in host metabolic homeostasis, although many of these relationships remain associative rather than causal. This review provides an integrated overview of antibiotic-driven gut microbiome dysbiosis, emphasizing the ecological, functional, metabolic, and resistome-level consequences of antibiotic exposure together with the mechanisms governing microbiome recovery. Current microbiome-targeted restoration strategies, including probiotics, phage therapy, fecal microbiota transplantation, and next-generation microbiome therapeutics, are critically evaluated with particular attention to their evidence maturity, limitations, and translational potential. Finally, key knowledge gaps and future research priorities are discussed to support the development of more effective microbiome-preserving antimicrobial strategies and to limit the long-term dissemination of antimicrobial resistance.

## 1. Introduction

The human gastrointestinal tract hosts a highly diverse and metabolically active microbial ecosystem that plays a central role in maintaining host homeostasis [1]. This complex microbial community, known as the gut microbiota, has garnered increasing scientific interest due to its extensive impact on human health and disease. As the microbiota co-develops with the host throughout life, it is involved in crucial physiological functions, including immune system maturation, pathogen defense, modulation of nutrient absorption and metabolism, and biosynthesis of essential compounds [2,3,4]. Among the various microbial niches in the human body, the gut microbiota represents the largest and most functionally diverse fraction of the human microbiome. Gut microbiota interactions have been recognized as one of the most transformative insights in modern biomedical research, especially given their essential roles in innate and adaptive immunity, neurotransmission, and chronic inflammation [5,6,7,8].

Advances in molecular biology, particularly in metagenomic sequencing, have significantly expanded our ability to analyze these microbial ecosystems in ways previously impossible. By enabling comprehensive profiling of microbial taxa, these technologies have facilitated correlations between microbiome composition and specific health or disease states [9,10]. The application of high-throughput sequencing has provided key insights into the “fingerprint” of the gut microbiota under varying physiological conditions, highlighting its role in gut barrier integrity, digestion, metabolic regulation, and immune homeostasis [7,11]. Furthermore, the gut microbiome’s metabolic potential is deeply intertwined with human physiology, influencing systemic processes such as energy metabolism, calcium and iron absorption, and lipid homeostasis [12]. Recent advancements in microbiome research have also linked the gut microbiome to circadian rhythmicity, nutritional responses, and detoxification [4,13].

Despite its notable stability and resilience, the gut microbiota is highly susceptible to environmental and pharmacological disturbances, particularly antibiotics [9,14]. Although antibiotics have undoubtedly revolutionized modern medicine by significantly reducing morbidity and mortality associated with bacterial infections, their widespread use has raised concerns regarding unintended effects on microbial homeostasis [8,9]. Antibiotics eliminate pathogenic bacteria but also disrupt commensal microbial communities, often leading to dysbiosis. Such imbalances are characterized by reduced microbial diversity, functional alterations, and the proliferation of opportunistic pathogens [15]. Dysbiosis has been implicated in a range of adverse health outcomes, including antibiotic-associated diarrhea (AAD), pseudomembranous colitis, and an increased risk of secondary infections, particularly those caused by *Clostridioides difficile* [16,17,18,19].

Additionally, the selective pressure exerted by antibiotics contributes to the emergence and dissemination of antimicrobial resistance (AMR), a major global health challenge. The persistence of antibiotic-resistant genes (ARGs) within the gut microbiota has been further linked to an enhanced likelihood of resistance transfer to pathogenic bacteria, compromising treatment efficacy and increasing infection risk [1,8,20,21]. In addition to direct antibiotic exposure, sub-therapeutic antibiotic use in the agricultural, livestock, and food sectors has exacerbated disruptions to the microbiome, contributing to chronic, low-dose exposure in human populations. Long-term microbial changes brought about by this constant selective pressure may predispose individuals to metabolic, inflammatory, and neurodegenerative disorders [9,22].

Even though numerous reviews have tackled individual aspects of gut microbiome dysbiosis [4,10,19,23,24,25,26,27,28,29,30], prior publications have mostly focused on compositional changes, specific disease associations, or isolated therapeutic approaches. Nonetheless, antibiotic-induced disturbances should be interpreted within a broader framework that integrates microbiome resilience, metabolic dysfunction, resistome remodeling, and strategies for microbiome restoration. In particular, recent developments in metagenomics, multi-omics approaches, and systems biology emphasize the importance of microbial interactions, horizontal gene transfer, and functional recovery mechanisms that extend beyond taxonomic alterations alone [31,32,33]. In this context, an updated, integrative synthesis is warranted to address this research gap, connect these related processes, and provide a comprehensive perspective on the consequences of antibiotic exposure.

Unlike previous reviews that have primarily summarized antibiotic-induced taxonomic changes or individual microbiome restoration strategies, this review adopts an integrative perspective linking ecological disruption, resistome remodeling, metabolic dysfunction, and microbiome recovery within a unified conceptual framework. Rather than considering these processes independently, we emphasize how alterations in microbial community resilience, horizontal gene transfer, and microbial metabolism determine the persistence of dysbiosis and influence the effectiveness of microbiome-targeted restoration strategies.

Given the intricate interplay between the gut microbiome and host health, understanding the full extent of antibiotic-induced perturbations is critical. Accordingly, this review integrates current evidence on antibiotic-induced ecological disruption, resistome dynamics, metabolic dysfunction, and microbiome restoration to provide a systems-level perspective on the mechanisms underlying persistent dysbiosis and recovery following antibiotic exposure. Moreover, it highlights current knowledge gaps and emerging therapeutic approaches to promote microbiome resilience and mitigate the long-term effects of antibiotic exposure.

## 2. Literature Search Methodology

This narrative review was developed through a comprehensive literature search ofmajor scientific databases (e.g., PubMed, ScienceDirect, and Web of Science). Relevant articles published primarily between 2016 and 2026 were retrieved using combinations of keywords including “gut microbiome,” “antibiotics,” “antibiotic-induced dysbiosis,” “gut resistome,” “resistome dynamics,” “antimicrobial resistance,” “mobile genetic elements,” “horizontal gene transfer,” “microbial metabolism,” “short-chain fatty acids,” “bile acids,” “microbiome resilience,” “microbiome restoration,” “probiotics,” “prebiotics,” “postbiotics,” “fecal microbiota transplantation,” and “phage therapy.” Search terms were refined through preliminary searches, and duplicate records were excluded during the screening process. Earlier landmark studies were retained when considered essential for providing historical context and supporting fundamental concepts in microbiome ecology and antimicrobial resistance.

Preference was given to systematic reviews, meta-analyses, randomized controlled trials, longitudinal cohort studies, and mechanistic investigations that provided clinically or biologically relevant insights into antibiotic-associated microbiome alterations and restoration strategies. Both clinical and preclinical studies were considered when they provided important mechanistic insights. Publications not available in English, studies with limited relevance to the review objectives, and reports lacking sufficient methodological detail were not included. The collected literature was critically evaluated and synthesized to provide an updated overview of antibiotic-associated gut microbiome dysbiosis, resistome dynamics, metabolic consequences, and emerging microbiome-targeted therapeutic approaches.

## 3. Healthy Gut Microbiota Composition

The healthy human gut microbiota contains approximately 3.8 × 10^13^ microbial cells, comprising bacteria, archaea, fungi, viruses, parasites, and yeasts [34,35]. These microorganisms encode more than 3.3 million non-redundant microbial genes, which generate thousands of metabolites and substantially expand the host metabolic repertoire [2,12,36,37,38]. Each individual possesses a unique gut microbial profile that evolves throughout life [9]. Consequently, microbiota-associated functions, including nutrient metabolism, maintenance of gut mucosal barrier integrity, immunomodulation, and protection against pathogens, depend not only on microbial composition but also on the functional capacity of the microbial community [36].

**Figure 1:**
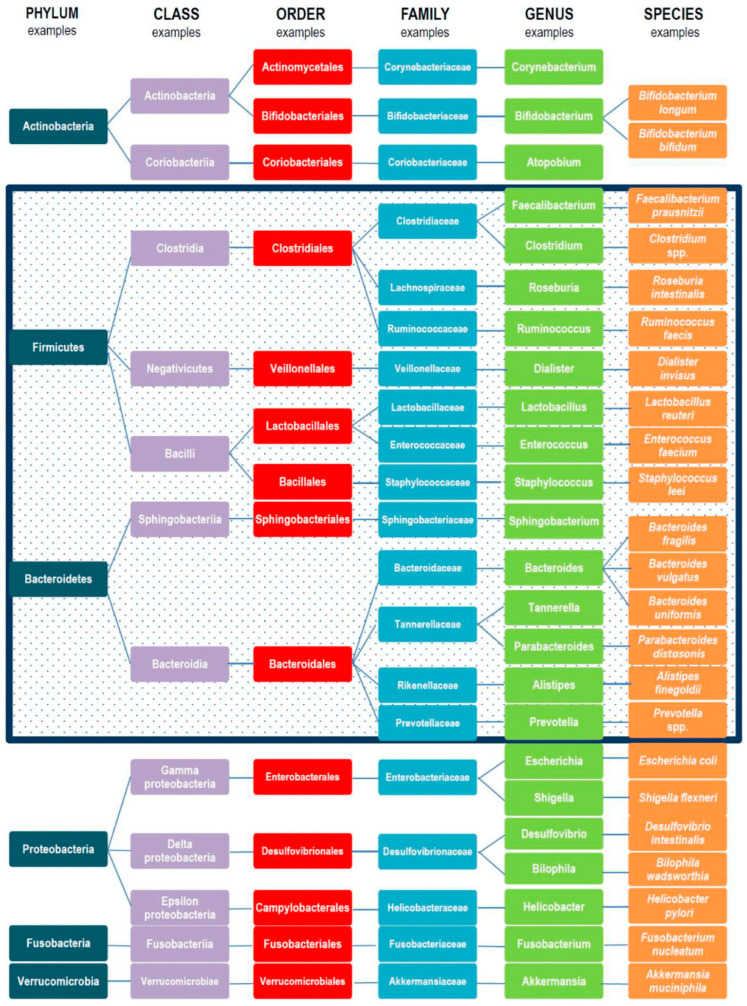
Taxonomic organization and representative members of the healthy human gut microbiota. The gut microbiota is predominantly composed of bacteria from the phyla Bacillota and Bacteroidota, which together account for more than 90% of the intestinal microbial community. Additional phyla, including Actinobacteria, Pseudomonadota, Fusobacteria, and Verrucomicrobia, contribute to microbial diversity and metabolic functionality. Among the representative taxa shown, *Faecalibacterium*, *Roseburia*, and *Ruminococcus* are recognized as major short-chain fatty acid producers; *Bifidobacterium*, *Lactobacillus*, and *Akkermansia* are representative taxa involved in immune modulation and maintenance of intestinal barrier function, whereas *Escherichia coli*, *Enterococcus faecalis*, *Staphylococcus aureus*, and *Helicobacter pylori* are included as representative opportunistic or pathogenic species. Because many gut microorganisms perform multiple biological functions, these functional assignments indicate their predominant reported roles. Reprinted from an open-access source [36].

Owing to technological advancements, researchers have been able to phylogenetically characterize and quantify gut microbiota by directly analyzing nucleic acids (DNA and RNA) extracted from stool samples. Sequencing of the 16S rRNA gene has emerged as a pivotal method for assessing bacterial diversity and abundance, allowing scientists to identify and classify gut microbial taxa [34,36,37]. Using these techniques, it has been established that most gut bacteria belong to four major phyla: Bacteroidota (Bacteroidetes), Bacillota (Firmicutes), Pseudomonadota (Proteobacteria), and Actinomycetota (Actinobacteria), while other phyla (e.g., Fusobacteria, Cyanobacteria, and Verrucomicrobia) occur at much lower abundance [2,5,12,16,36,39] (Figure 1). Bacillota and Bacteroidota together account for more than 90% of the gut microbiota [2,12]. Although alterations in the Bacillota/Bacteroidota ratio have been reported across different physiological and pathological conditions, this metric is no longer considered a reliable standalone marker of gut health or dysbiosis and should be interpreted within the broader ecological and functional context of the microbiome [2,12,40]. Bacillota encompass more than 200 genera, including *Lactobacillus*, *Bacillus*, *Clostridium*, *Enterococcus*, and *Ruminococcus*, with *Clostridium* species representing the majority. Bacteroidota primarily include the genera *Bacteroides* and *Prevotella* [36]. Actinomycetota are mainly represented by *Bifidobacterium* and *Collinsella* [5].

Increasing evidence indicates that microbiome health should not be interpreted solely in terms of taxonomic composition but also in terms of functional stability, ecological resilience, and metabolic adaptability. In this context, the concepts of “healthy microbiome” and “dysbiosis” remain incompletely standardized, as microbial communities are continuously shaped by host physiology, dietary habits, environmental factors, and microbial interactions [41]. Functional redundancy has emerged as a central ecological property of the gut microbiome, referring to the capacity of phylogenetically distinct microorganisms to perform overlapping metabolic functions, thereby preserving ecosystem stability despite compositional fluctuations [42,43]. Healthy microbiomes generally exhibit high functional redundancy and network resilience, whereas disease-associated states are frequently characterized by reduced ecological stability, disrupted metabolic interdependencies, and altered keystone microbial functions [44,45]. Importantly, recent metagenomic and metaproteomic studies have demonstrated that significant functional alterations can occur even in the absence of major taxonomic changes, emphasizing the limitations of purely taxonomy-based interpretations of microbiome health [43,46].

Longitudinal dietary intervention studies further showed that healthy gut microbiota often maintains substantial compositional and metabolic resilience despite short-term nutritional perturbations, although responses remain highly individualized according to baseline microbial configurations [47]. Similarly, ecological modeling approaches demonstrated that microbiome stability can be evaluated using both observational and systems-level ecological frameworks, reinforcing the concept of the gut microbiota as a dynamic yet resilient ecosystem [48]. Recent multi-omics investigations additionally highlighted that gut physiology, intestinal transit time, luminal pH, and environmental exposures significantly contribute to interindividual microbiome variability and metabolic outputs, underlining the importance of host–environment interactions in shaping microbiome functionality [49]. Recent ecological frameworks further emphasized the role of microbial keystone taxa, defined as microorganisms that exert disproportionately large effects on ecosystem structure, stability, and function relative to their abundance. These taxa contribute to microbiome resilience through metabolic specialization, ecological interactions, and maintenance of community homeostasis, demonstrating that low-abundance microorganisms may still exert critical ecosystem-level effects [50].

Beyond taxonomic composition, gut microbiota functionality is strongly linked to the production of short-chain fatty acids (SCFAs), particularly butyrate, a key metabolite involved in intestinal and systemic homeostasis. Butyrate-producing bacteria, including *Faecalibacterium*, *Anaerostipes*, *Agathobacter*, and *Roseburia* species, contribute to epithelial barrier integrity, mucus production, immune modulation, and anti-inflammatory signaling pathways [51,52]. Higher abundances of butyrate-producing taxa have also been associated with a reduced risks of infection-related hospitalization and mortality, supporting the clinical relevance of microbiome-derived metabolites in host resilience [53]. Moreover, dietary fibers selectively stimulate SCFA-producing taxa, although these responses are strongly influenced by baseline microbial composition and interindividual variability [51].

Emerging mechanistic studies have further supported the immunomodulatory role of key commensal taxa, particularly *Faecalibacterium prausnitzii*. Recent in vitro and ex vivo models have demonstrated that *F. prausnitzii* modulates epithelial–immune crosstalk, promotes anti-inflammatory signaling, and induces IL-10 production in intestinal and circulating immune cells, reinforcing its proposed role in gut homeostasis and resilience [54,55]. Similarly, *Akkermansia muciniphila* has emerged as another important commensal associated with gut barrier integrity, immune modulation, and SCFAs-mediated metabolic regulation. Current evidence suggests that *A. muciniphila* contributes to epithelial protection, tight junction maintenance, and anti-inflammatory signaling, further supporting the concept that specific keystone taxa may exert systemic effects extending beyond local gut ecology [56].

Contemporary studies have additionally shown that antibiotic exposure profoundly perturbs bacterial, phage, and metabolic components of the gut microbiome, although partial ecological resilience may occur over time [57]. Nevertheless, persistent reductions in SCFAs-producing taxa and metabolite alterations have been associated with long-term immune, neuroendocrine, and inflammatory consequences, particularly following early-life microbiome disruption [58].

In conclusion, these findings support a growing shift from descriptive taxonomic profiling toward function-oriented, systems-level characterization of the gut microbiome, emphasizing the importance of ecological resilience, microbial metabolites, and host–microbe interactions in maintaining intestinal and systemic homeostasis. Recent advances in microbiome ecology and systems biology contributing to the redefinition of healthy gut microbiome functionality are summarized in Table 1.

**Figure 2:**
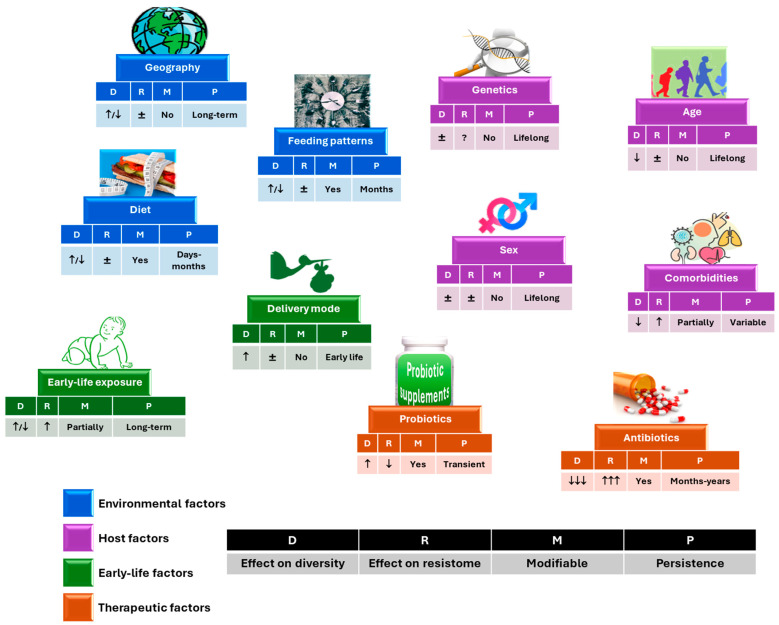
Major determinants of gut microbiome composition, resistome dynamics, and microbiome resilience. Environmental (blue), host-related (purple), early-life (green), and therapeutic (orange) factors influence the gut microbiome to varying degrees. For each determinant, the relative impact on microbial diversity (D), resistome composition (R), modifiability (M), and persistence of the associated effects (P) is summarized. Upward and downward arrows indicate positive or negative influences, respectively, while the number of arrows reflects the relative magnitude of the effect; “±” depicts variable or context-dependent effects; and “?” denotes insufficient evidence The figure highlights the particularly profound and long-lasting impact of antibiotics on microbial diversity and the enrichment of antibiotic resistance genes, in contrast to other determinants that exert more moderate, transient, or context-dependent effects. These factors collectively modulate microbial diversity, metabolic activity, immune maturation, and long-term microbiome stability. Created based on information from [60,61,62].

Current evidence indicates that gut microbiome composition is shaped by a complex interplay of intrinsic, environmental, dietary, and health-related factors (Figure 2), although their relative contributions and implications for microbiome-targeted interventions remain incompletely understood [60].

Among these factors, diet is one of the strongest modulators of microbiome composition and metabolic activity. Long-term dietary habits influence microbiome maturation and stability, whereas short-term dietary interventions may induce rapid compositional shifts within a few days, highlighting the therapeutic potential of dietary modulation [62,63,64]. In addition to dietary influences, age, host genetics, smoking, medication exposure, and physical activity also contribute substantially to microbiome variability, further complicating efforts to isolate the independent effects of specific environmental factors on disease susceptibility and progression [62]. Consequently, large-scale, well-phenotyped longitudinal cohorts integrating multi-omics and environmental data are increasingly recognized as essential for elucidating host–microbiome interactions and advancing personalized microbiome-based therapeutic strategies [60].

## 4. Early-Life Determinants of Gut Microbiome Resilience and Vulnerability to Antibiotic Disruption

Early life represents a critical developmental window during which gut microbial colonization contributes to immune education, epithelial maturation, metabolic programming, and long-term host–microbiota homeostasis [65,66]. Neonatal microbial assembly is shaped by maternal microbial reservoirs, mode of delivery, feeding practices, gestational age, environmental exposures, hospitalization, and antibiotic use [67,68]. These factors are particularly relevant to antibiotic-driven dysbiosis because they define baseline microbial diversity, functional redundancy, colonization resistance, and the capacity of the gut microbiome to recover after perturbation.

**Figure 3:**
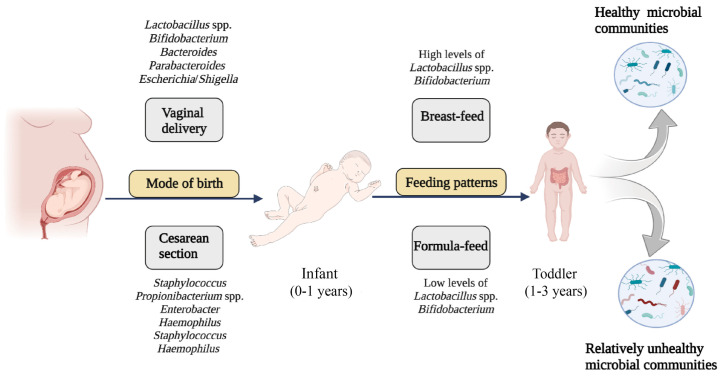
Early-life gut microbiota development is influenced by mode of delivery and infant feeding patterns. Vaginal delivery and breastfeeding promote colonization by beneficial anaerobic taxa, including *Bifidobacterium* and *Lactobacillus*, whereas cesarean delivery and formula feeding are associated with altered microbial succession and reduced abundance of commensal bacteria. Reprinted from an open-access source [85].

During infancy, gut microbiota maturation is highly dynamic and follows partially conserved trajectories, with early *Bifidobacterium*-rich communities progressively shifting toward more complex adult-like configurations dominated by Bacillota, Bacteroidota, and Actinomycetota [16,36,62,68,69,70]. Vaginal delivery and breastfeeding generally support maternal strain transmission and enrichment of beneficial anaerobic taxa, including Bifidobacterium and Bacteroides, whereas cesarean delivery, formula feeding, prematurity, hospitalization, and early antibiotic exposure are frequently associated with delayed microbial maturation, reduced diversity, increased Pseudomonadota abundance, and expansion of opportunistic microorganisms [71,72,73,74,75,76,77]. These ecological changes may affect colonization resistance, SCFA production, bile acid metabolism, mucosal immune maturation, and long-term susceptibility to immune, metabolic, respiratory, and infectious outcomes [23,78,79,80,81,82,83,84]. Figure 3 provides a concise overview of how delivery mode and feeding practices influence early microbiota development.

Early-life perturbations also influence resistome and mobilome development. Metagenomic studies indicate that delivery mode, gestational age, feeding practices, geography, hospitalization, and antibiotic exposure shape the infant resistome, with mobile genetic elements, plasmids, and bacteriophages contributing to ARG dissemination during microbiome maturation [24,86,87]. Cesarean delivery, prematurity, formula feeding, and antibiotic exposure have been associated with increased ARG burden and enrichment of ARG-carrying taxa such as *Escherichia coli* and *Klebsiella* spp., whereas breastfeeding and higher Bifidobacterium abundance may partially mitigate resistome expansion and support functional microbiome stability [24,86,87,88,89,90]. The main early-life factors relevant to antibiotic-associated dysbiosis and resistome expansion are summarized in Table 2.

Antibiotic exposure during pregnancy, delivery, and infancy is among the most disruptive influences on early gut microbiome development. Antibiotics used during pregnancy or intrapartum prophylaxis can cross the placental barrier, alter maternal microbial communities, disrupt vertical microbial transmission, and are thought to modulate the maternal–fetal metabolite pool [16,65,95]. Cesarean delivery, often accompanied by maternal antibiotic administration, may further impair microbial transfer and promote ARG expansion within the neonatal gut microbiome [96,97]. Perinatal and early-life antibiotic exposure typically reduces beneficial taxa such as Bifidobacterium and Bacteroides while enriching opportunistic microorganisms, including Pseudomonadota and *Clostridium* spp., thereby altering microbial succession and potentially increasing susceptibility to later immune, metabolic, respiratory, and neurodevelopmental disorders reported in observational and longitudinal studies [16,95,98].

**Figure 4:**
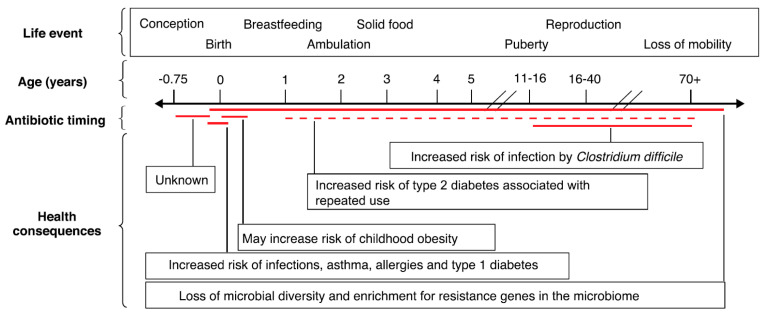
Health consequences of antibiotic-induced disruption of the gut microbiota during development and adulthood. Red lines—a single dose of antibiotics within the time period has been linked to a health consequence; dotted red lines—multiple doses of antibiotics within the time period are required to observe a link. Reprinted from an open-access source [95].

This vulnerability is particularly important in preterm infants, in whom antibiotic treatment is common and often lifesaving but occurs against a background of microbiome immaturity, hospitalization, invasive procedures, and neonatal intensive care unit-associated selective pressures. Preterm infants frequently exhibit reduced microbial diversity, delayed microbial maturation, depletion of beneficial anaerobes such as *Bifidobacterium* and *Lactobacillus*, increased Pseudomonadota dominance, and colonization by opportunistic or multidrug-resistant organisms, including *Enterococcus*, *Klebsiella*, *Pseudomonas*, *Staphylococcus*, and *Candida* [16,36,76,77,99,100,101]. Prolonged or aggressive antibiotic regimens further intensify these disturbances and have been associated with necrotizing enterocolitis, late-onset sepsis, mortality, persistent microbiome instability, transient ARG expansion, and enrichment of potentially pathogenic taxa such as *Klebsiella*, *Enterococcus*, *Staphylococcus*, *K. pneumoniae*, and *E. coli* [28,95,97,98,102,103,104,105,106,107,108,109,110,111]. These findings emphasize the need to balance infection control with microbiome preservation in neonatal care [95,108]. Figure 4 summarizes the health consequences associated with antibiotic-induced gut dysbiosis across developmental stages.

More broadly, antibiotic-associated dysbiosis can predispose individuals to clinically relevant complications across life stages. One of the most severe examples is *Clostridioides difficile* infection, which arises when antibiotic-mediated microbiome depletion permits *C. difficile* colonization and toxin production [14,112]. Antibiotic-associated diarrhea is also common, affecting approximately 5–35% of antibiotic-treated individuals, while cumulative antibiotic exposure is associated with progressively increasing risk of antibiotic-associated adverse events [112,113,114]. Beyond gastrointestinal complications, antibiotic-induced dysbiosis has been associated with impaired gut barrier integrity, altered immune signaling, and metabolic dysfunction, while emerging evidence suggests potential links with immune-mediated disorders [9].

Assessment of antibiotic-associated dysbiosis increasingly relies on integrated approaches combining 16S rRNA gene sequencing, shotgun metagenomics, microbial diversity indices, resistome profiling, microbial metabolite measurements such as SCFAs, and inflammatory or barrier-related biomarkers, including fecal calprotectin and zonulin [115,116,117]. Together, these approaches provide a more comprehensive view of the transition from eubiosis to dysbiosis and help clarify how antibiotic exposure alters microbial composition, functional capacity, resistome architecture, and long-term microbiome resilience.

Overall, early-life and perinatal factors are retained in this review only as contextual modifiers of antibiotic-induced dysbiosis. They help explain why the magnitude, persistence, and clinical consequences of antibiotic-related microbiome disruption differ across individuals, particularly in infants, preterm neonates, and other vulnerable populations. The following sections therefore focus on the direct ecological, resistome-level, metabolic, and clinical consequences of antibiotic exposure and on strategies aimed at microbiome restoration.

## 5. Influence of Antibiotics on the Human Microbiome

Antibiotics have long been the cornerstone of bacterial infection management; however, their widespread use profoundly affects gut microbiota homeostasis and challenges the delicate microbial balance required for host health [9]. Although administered to eliminate pathogenic microorganisms, antibiotics also alter the composition, diversity, and functional activity of commensal microbial communities, with effects that may persist for months or, in some cases, become long-lasting or irreversible [2,14,118].

Antibiotic-induced dysbiosis is characterized by the depletion of beneficial microbial taxa, the expansion of opportunistic pathogens, and the disruption of microbial metabolic and immunomodulatory functions, thereby influencing host immunity, metabolism, and intestinal barrier integrity [9]. The extent of gut microbiota disruption depends on multiple factors, including antibiotic spectrum, duration of exposure, route of administration, and mechanism of action, as different antimicrobial classes selectively suppress or enrich distinct microbial populations [36]. Consequently, antibiotic exposure contributes not only to compositional shifts within the gut microbiota but also to resistome expansion and altered microbial resilience. The following subsections summarize the major antibiotic-associated changes in gut microbial composition and the emergence of AMR within the human microbiome.

### 5.1. Antibiotic-Induced Alterations in Gut Microbiota

The impact of antibiotics on the gut microbiota is highly heterogeneous and depends on multiple factors, including antibiotic class, spectrum of activity, dosage, treatment duration, host age, genetic background, diet, and concomitant medications [9]. Broad-spectrum antibiotics appear to cause the most profound alterations, marked by reductions in microbial diversity, disruption of the Bacillota/Bacteroidota balance, depletion of beneficial commensals, and expansion of opportunistic microorganisms [16,36]. Beyond taxonomic changes, these perturbations may compromise intestinal homeostasis by altering epithelial proliferation, apoptosis, immune signaling, and enzymatic activity, while also promoting the release of intracellular microbial products associated with gut dysregulation [1].

Although this review primarily focuses on the intestinal microbiome, antibiotic exposure also affects other host-associated microbial ecosystems, including the oral, pulmonary, skin, and vaginal microbiota [119]. Such disturbances may contribute to recurrent infections, dermatitis, bacterial vaginosis, and fungal overgrowth. One of the best-known examples is the reduction of vaginal microbial diversity following broad-spectrum antibiotic therapy, which facilitates overgrowth of opportunistic fungi such as *Candida albicans* [120]. These findings emphasize that antibiotic-associated dysbiosis should not be regarded as an isolated intestinal phenomenon, but rather as a systemic ecological disturbance affecting multiple interconnected microbial niches.

Importantly, microbiota recovery after antibiotic exposure is often incomplete and highly individualized. While healthy young adults may partially recover their baseline microbial composition after treatment ends, restoration is frequently delayed or impaired in older individuals due to immunosenescence, metabolic alterations, reduced ecological resilience, and polypharmacy-related drug–microbiome interactions [2,27,121]. Recent longitudinal and metagenomic investigations further demonstrated that antibiotic-induced dysbiosis may persist for months or even years after therapy. Karimianghadim et al. [122] reported prolonged depletion of beneficial taxa, including *Bifidobacterium bifidum* and *A. muciniphila*, along with persistent alterations in microbial functional pathways and bacteriophage populations. Similarly, Ng et al. [123] highlighted that microbiota recovery is strongly influenced by environmental reservoirs, dietary factors, and community context; furthermore, that reduced ecological resilience delays restoration following antibiotic exposure.

Additional longitudinal analyses showed that although microbial richness may partially recover after short antibiotic courses, persistent alterations in taxonomic composition, resistome architecture, and metabolic activity often remain detectable long after treatment cessation, a phenomenon increasingly referred to as “antibiotic scarring” [124]. Population-scale metagenomic studies have further indicated that prior exposure to clindamycin, fluoroquinolones, and broad-spectrum penicillins may remain associated with reduced microbial diversity and altered community structure for several years [125]. Nevertheless, the extent and long-term persistence of these alterations remain incompletely understood, as many studies rely on heterogeneous sequencing methodologies, variable follow-up durations, and limited functional validation, complicating direct comparisons between cohorts.

The metabolic consequences of antibiotic-induced dysbiosis arise from the disruption of microbial functions that maintain intestinal homeostasis. In particular, the depletion of obligate anaerobic commensals and SCFA-producing bacteria compromises key metabolic pathways involved in epithelial barrier maintenance, bile acid transformation, and immune regulation, thereby linking ecological disruption to systemic host responses. Beyond ecological disruption, antibiotic-induced dysbiosis also has profound metabolic consequences. Reduced microbial diversity has been associated with altered plasma and fecal metabolomic profiles involving amino acid, carbohydrate, lipid, and bile acid metabolism [126]. In particular, depletion of SCFA-producing bacteria may reduce acetate, propionate, and butyrate production, thereby impairing epithelial barrier integrity, immune regulation, and systemic metabolic homeostasis. Experimental evidence further suggests that these microbiota-related metabolic disturbances may contribute to inflammatory and cardiovascular complications by altering host–microbe metabolic signaling [127]. Emerging multi-omics studies also indicate that microbiota-derived metabolites and bile acid signaling pathways may influence host transcriptional and epigenetic regulation, highlighting complex bidirectional interactions between microbial communities and host gene expression programs [128].

Increasing evidence also suggests that the clinical consequences of dysbiosis extend beyond gastrointestinal disorders and may influence responses to advanced therapies. Broad-spectrum antibiotic exposure has been linked to depletion of metabolically active commensals, reduced SCFA production, systemic metabolomic alterations, and impaired CAR-T immunotherapy efficacy, underscoring the importance of microbiota integrity in modulating systemic immune responses and therapeutic outcomes [129]. Notably, microbiota disruption is not limited to antibiotics. Several non-antibiotic drugs have also been shown to impair colonization resistance, alter microbial interactions, and facilitate the expansion of enteropathogens, suggesting that pharmacological perturbation of the microbiome may represent a broader, underrecognized contributor to disease susceptibility [130].

Antibiotic-induced dysbiosis may further impair colonization resistance and host immune defenses, thereby promoting the expansion of opportunistic and MDR pathogens. Experimental and clinical studies have demonstrated that depletion of the commensal microbiota following broad-spectrum antibiotic exposure facilitates pathogen overgrowth, including carbapenem-resistant Enterobacteriaceae (CRE), and increases susceptibility to invasive fungal infections by altering microbial metabolites and impairing IL-17-mediated antifungal immunity [131,132]. Recent evidence also links colonization by MDR organisms to reduced microbial diversity, depletion of beneficial commensals, expansion of pathobionts such as *Enterococcus* and *Klebsiella*, and broader immune dysregulation characterized by altered cytokine profiles and inflammatory signaling [29].

Finally, dysbiosis-associated depletion of butyrate-producing bacteria has also been implicated in extraintestinal disorders, including recurrent urinary tract infections, in which disruption of the gut–bladder axis may contribute to altered immune responses and increased susceptibility to bacterial colonization [133]. These findings indicate that antibiotic-induced dysbiosis extends far beyond transient taxonomic alterations, representing a complex ecological and functional disturbance with metabolic, immunological, and therapeutic implications across multiple organ systems.

Table 3 summarizes the ecological, metabolic, immunological, resistome-related, and clinical consequences of the major antibiotic classes, illustrating how antibiotic exposure disrupts the gut microbiome across multiple interconnected biological levels.

### 5.2. Antibiotic Resistance

Antibiotic administration profoundly reshapes the gut microbiota and resistome by altering microbial composition, selecting for resistant taxa, and promoting the dissemination of ARGs. Broad-spectrum antibiotic exposure rapidly increases ARG abundance, facilitates horizontal gene transfer (HGT) among commensal and pathogenic bacteria, and contributes to the emergence of MDR organisms [135,136]. The gut microbiota, therefore, serves as a major reservoir of resistance determinants, particularly tetracycline, beta-lactam, and macrolide resistance genes, which may persist and co-select unrelated ARGs under continued selective pressure [136]. Interestingly, although antibiotic exposure frequently increases overall resistome burden, taxonomic depletion may paradoxically reduce resistome diversity by eliminating susceptible microbial populations [137].

Recent metagenomic and longitudinal studies further demonstrate that antibiotic-associated resistome remodeling is highly dynamic and shaped by ecological context, microbial interactions, and MGEs. Population-level analyses identified strong associations between antibiotic consumption and ARG burden across human gut microbiomes, particularly for multidrug, tetracycline, macrolide, and beta-lactam resistance determinants [135]. Similarly, Zhou et al. [136] reported marked regional and taxonomic variability in gut resistomes, highlighting the role of MGEs, transposases, and recombinases in ARG dissemination. Mechanistically, HGT has emerged as a central driver of resistome evolution. Genome-scale comparative analyses indicate that different classes of mobile genetic elements contribute unequally to ARG dissemination. Transposable elements appear to represent the predominant carriers of resistance genes across bacterial taxa, whereas integrons frequently facilitate co-selection through hitchhiking with other MGEs. Computational frameworks additionally suggest that plasmids, bacteriophages, and insertion sequences contribute through complementary mobilization pathways rather than acting as equivalent vectors of ARG dissemination, emphasizing that resistome evolution results from the interplay of multiple MGE classes rather than a single dominant mechanism [138,139,140,141]. Human metagenomic analyses indicate that antibiotic exposure preferentially enriches mobile rather than non-mobile ARGs, with co-selection occurring predominantly through class 1 integrons on plasmids. However, large-scale comparative analyses also suggest that successful horizontal transfer is constrained by host compatibility and ecological context, as several clinically relevant carbapenemase and cephalosporin resistance genes remain taxonomically restricted despite residing on mobilizable plasmids [142,143]. Long-read longitudinal metagenomic analyses further suggest that bacteriophages actively contribute to genome plasticity and horizontal gene transfer through interactions with bacterial insertion sequences, indicating that phage-mediated mobilization may represent an additional mechanism sustaining resistome evolution beyond plasmid-mediated transfer [144].

Large population-based metagenomic analyses indicate that antibiotic-associated microbiome perturbations may persist for several years after treatment, with prior exposure to clindamycin, fluoroquinolones, and flucloxacillin remaining associated with long-lasting alterations in bacterial community composition, suggesting that ecological recovery may remain incomplete long after antibiotic withdrawal [125]. Longitudinal human metagenomic studies similarly indicate that resistome recovery may lag behind taxonomic recovery following antibiotic exposure. Persistent plasmid-associated ARGs and core resistance determinants have been detected despite partial restoration of bacterial diversity, while longitudinal analyses of the infant plasmidome showed that plasmid dynamics only partially mirror changes in bacterial community composition, supporting the concept that mobile genetic elements represent an independent ecological layer contributing to long-term resistome persistence [145,146,147]. Complementary genome-scale comparative analyses further demonstrate that integrons, transposons, insertion sequences, and specific plasmid replicons contribute differently to ARG mobilization, highlighting the complementary roles of distinct MGEs in shaping resistome evolution [148].

Antibiotic-induced dysbiosis was also associated with the expansion of opportunistic pathobionts, including *Enterococcus* and *Escherichia*, as well as the enrichment of multidrug and efflux-mediated resistance determinants [137]. Nevertheless, recent clinical evidence suggests that resistome responses may vary with treatment duration and ecological resilience, as shorter and prolonged antibiotic regimens do not always produce major differences in ARG abundance or microbial diversity [149].

Beyond transient ARG enrichment, accumulating evidence indicates that antibiotic-associated resistance may persist long after treatment cessation. Hansen et al. [150] demonstrated that enteric infections were associated with increased ARG diversity and enrichment of extended-spectrum beta-lactamase (ESBL) genes, some of which persisted after clinical recovery, suggesting sustained ecological disruption of the gut resistome. At the population level, Rahman et al. [151] further showed that increases in antibiotic consumption were associated with prolonged increases in AMR across European countries, with resistance trends persisting for several years after antibiotic use escalated. Complementing these observations, Baek et al. [152] identified distinct antimicrobial-resistant bacterial populations with differential resistome profiles and proposed that extensively acquired antimicrobial-resistant bacteria may actively shape post-antibiotic microbiome composition and promote the persistence of antimicrobial resistance.

Recent observational and multi-omics studies have linked gut resistome remodeling with alterations in lipid metabolism and glucose homeostasis. Lee et al. [153] associated changes in the gut resistome with dyslipidemia, depletion of SCFA-producing bacteria, and enrichment of pro-inflammatory ARG-associated taxa, whereas Wu et al. [154] identified extensive microbiome–metabolome interactions associated with impaired glucose homeostasis in type 2 diabetes. These findings suggest that microbiome and resistome alterations may contribute to systemic metabolic and inflammatory disturbances, although the underlying mechanisms and causal relationships remain to be fully established.

**Figure 5:**
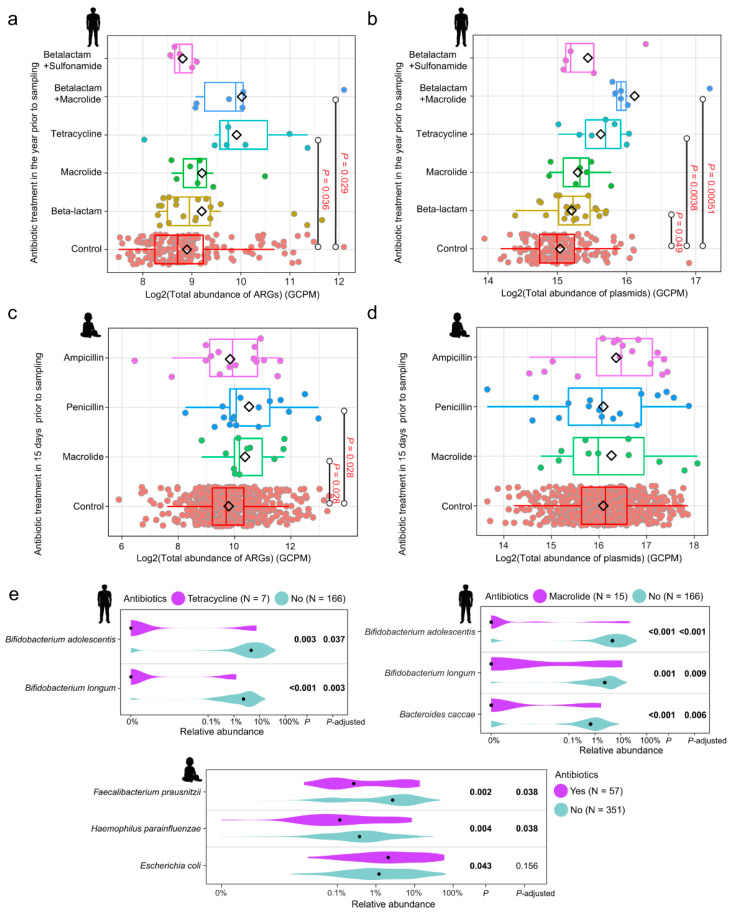
The effects of different antibiotics on ARG and plasmid abundance, as well as on the relative abundance of bacterial species. Changes in (**a**) ARG abundance and (**b**) plasmid abundance in the gut of adults who had taken one of five major antibiotics or antibiotic combinations in the year before sampling. Changes in (**c**) ARG abundance and (**d**) plasmid abundance in the gut of infants who had taken one of three major antibiotics in the 15 days before sampling. (**e**) Members of the 20 most abundant bacterial species whose abundance in the gut differed significantly between (**top**) adults who had taken tetracycline or macrolide in the year before sampling and those who had not received antibiotic treatment, and (**bottom**) infants who had taken antibiotics (mixed effects) in the 15 days before sampling and those who had not within the first year. Reprinted from an open-access source [145].

Figure 5 summarizes the effects of different antibiotics on ARG abundance, plasmid dissemination, and relative bacterial abundance in the gut microbiome. Although ARG levels may gradually decline after antibiotic cessation, resistance traits can persist within microbial communities, particularly under continued low-dose selective pressures [151,152]. Microbiome resilience after antibiotic exposure appears to depend on multiple ecological factors, including species diversity, functional redundancy, and colonization resistance against opportunistic pathogens [139,149]. These findings indicate that the gut microbiome functions not only as a passive reservoir of ARGs, but also as an active ecological regulator of ARG persistence, dissemination, and HGT. Consequently, microbiome-targeted approaches aimed at restoring microbial diversity and modulating microbiome–pathogen interactions are increasingly explored as complementary strategies to mitigate AMR spread [26,155].

## 6. Strategies for Reshaping/Correcting Gut Microbiota

**Figure 6:**
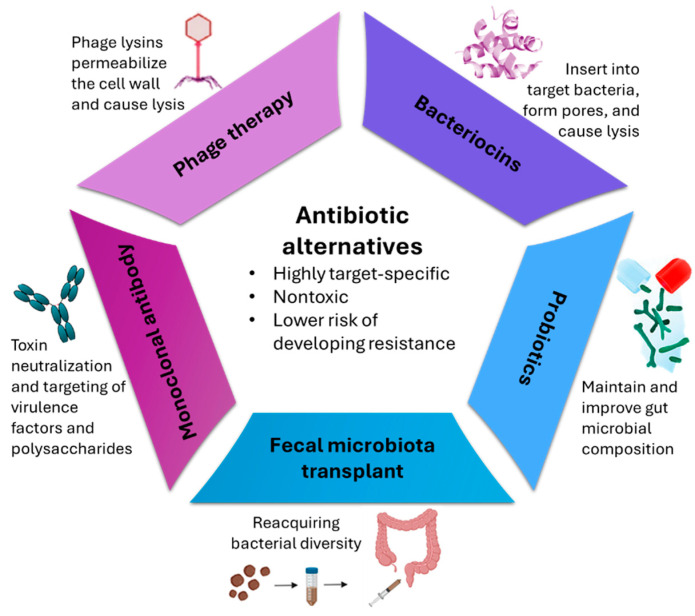
Various alternatives to antibiotics can be used alone or in combination with antibiotic treatment. Adapted from an open-access source [16].

As described above, antibiotic treatment significantly alters the gut microbiota, disrupting microbial balance and reducing diversity. To reduce the risk of antibiotic-associated disorders, strategies to restore microbial equilibrium after antibiotic treatment are required [14,69]. Several interventions have been explored to facilitate microbiota recovery, including the administration of probiotics, FMT, phage therapy, bacteriocins, and monoclonal antibodies [6,16,39] (Figure 6). The main characteristics of current microbiota restoration therapies are summarized in Table 4.

### 6.1. Probiotics, Prebiotics, and Postbiotics

Probiotics are beneficial microorganisms that may support recovery of the gut microbiota following antibiotic-induced dysbiosis; however, accumulating evidence indicates that their effects are highly strain-, host-, and context-dependent rather than universally restorative [160]. Antibiotic treatment disrupts microbial diversity, depletes beneficial commensals, and facilitates colonization by opportunistic pathogens, increasing susceptibility to conditions such as AAD and CDI [16,112]. Mechanistically, probiotics may stabilize microbial communities, reinforce epithelial barrier integrity, compete with pathogens for ecological niches, produce antimicrobial peptides and bacteriocins, and modulate host immune responses [160,161,162]. Clinical evidence supports the use of selected probiotic strains for specific indications, particularly AAD prevention and, to a lesser extent, recurrent CDI, whereas evidence for broader microbiome restoration remains inconsistent across strains, patient populations, and clinical settings [14,112].

Metagenomic and clinical studies have further suggested that probiotic-mediated microbiota recovery is considerably more heterogeneous than initially assumed. Although several studies reported improvements in microbial diversity and resistome modulation, responses appear strongly dependent on strain selection, baseline microbiota composition, and ecological context. FitzGerald et al. [163] observed only modest microbiota recovery during *Helicobacter pylori* eradication therapy despite successful but transient engraftment of administered strains, whereas John et al. [164] reported preservation of microbial diversity together with reduced Enterobacteriaceae abundance and lower ARG levels after probiotic supplementation. Similarly, synbiotic-based interventions promoted recovery of butyrate-producing bacteria, increased SCFAs production, and improved gut barrier integrity following antibiotic exposure [165], suggesting that functional and metabolic recovery may be as important as taxonomic restoration. In contrast, Montassier et al. [166] demonstrated marked person-specific responses, showing that probiotics reduced ARG abundance in some individuals while promoting resistome expansion and resistant taxa in others. Comparable variability was reported in studies investigating decolonization of antimicrobial-resistant pathogens, in which probiotics and prebiotics improved colonization resistance in selected settings [167], while concerns regarding the persistence or transfer of resistance determinants persisted [167]. Together, these findings indicate that microbiome-targeted interventions act more as context-dependent ecological modulators than as universally restorative therapies, emphasizing the need for strain-level characterization and personalized approaches.

Interest has therefore shifted toward next-generation probiotics and function-oriented microbiome therapies. Species such as *A. muciniphila* and *F. prausnitzii* are increasingly investigated for their effects on epithelial barrier integrity, immune signaling, host metabolism, and SCFA production rather than for simple restoration of bacterial diversity [168]. Experimental and clinical studies have further suggested that pasteurized bacterial formulations, synbiotic combinations, and metabolite-oriented interventions may provide greater stability and more reproducible effects than conventional probiotics alone [169]. Similarly, next-generation synbiotic formulations enriched with SCFA-producing taxa and targeted prebiotics promoted persistence of beneficial strains and metabolic recovery within gut ecosystems [170]. More advanced approaches, including engineered microbial delivery systems designed to improve intestinal colonization and anti-inflammatory activity, are also being explored, although their clinical applicability remains uncertain [171].

At the same time, increasing evidence supports the development of personalized microbiome modulation strategies. Baseline microbiota composition and host-related characteristics appear to substantially influence probiotic colonization efficiency and therapeutic response [172]. Large-scale microbiome analyses have further demonstrated that pre-existing microbial community structure strongly affects *Bifidobacterium* persistence after supplementation, supporting the concept that future probiotic interventions may need to be individualized according to host-specific microbiome features rather than applied uniformly across populations [173].

Besides probiotics, prebiotics and postbiotics also represent promising approaches for microbiota restoration. Prebiotics are selectively fermentable substrates, including inulin, fructooligosaccharides, galactooligosaccharides, and polyphenols, that stimulate the growth and activity of beneficial microorganisms such as *Lactobacillus*, *Bifidobacterium*, *Akkermansia*, *Roseburia*, and *Faecalibacterium*. Through enhanced SCFA production and modulation of microbial metabolism, prebiotics contribute to immune regulation, anti-inflammatory effects, and maintenance of gut barrier integrity [174,175]. In parallel, postbiotics, defined as bioactive metabolites and structural components generated during microbial fermentation, including peptides, microbial cell fractions, and metabolic byproducts, exert immunomodulatory, antimicrobial, and anti-inflammatory effects. Compared with live probiotics, postbiotics may offer improved stability and safety profiles, making them attractive candidates for microbiome-directed therapies [174,176].

Overall, probiotics, prebiotics, and postbiotics represent complementary microbiome-targeted strategies rather than universally effective therapies. Current evidence indicates that successful restoration after antibiotic exposure depends not only on reintroducing beneficial taxa but also on host-specific microbial ecology, ecological resilience, metabolic recovery, and the functional re-establishment of the intestinal ecosystem. Further well-designed longitudinal and mechanistic studies are required to determine which interventions are effective, for whom, and under which clinical conditions.

### 6.2. Phage Therapy

Another promising method for modifying the gut microbiota and reversing antibiotic-induced dysbiosis is phage therapy. Bacteriophages (phages) are viruses that specifically infect and lyse bacteria, making them effective antibacterial agents with great potential to restore microbial balance [9,177]. Unlike antibiotics, which often disrupt both pathogenic and commensal microorganisms, phages exhibit high host specificity, allowing selective pathogen depletion while largely preserving beneficial microbial communities [178]. Increasing evidence also suggests that phages may influence gut ecology beyond direct bacterial killing. Alterations in the intestinal phageome have been associated with gastrointestinal and liver diseases, while selective depletion of pathobionts improved inflammatory and metabolic phenotypes in preclinical models of IBD and non-alcoholic fatty liver disease [179].

Recent translational studies indicate that phage therapy may function as a form of precision microbiota editing rather than solely as an antibacterial intervention. Encapsulated phage delivery systems engineered for improved intestinal stability and colon-specific release selectively eradicated *Salmonella*, reduced inflammatory cytokine production, and limited antibiotic-associated dysbiosis while preserving commensal microbiota in murine colitis models [180]. Similarly, targeted phage therapy against *Salmonella*-associated intestinal inflammation improved immune and metabolic parameters and reduced cytokine expression, supporting a broader immunomodulatory role of bacteriophages within dysbiotic gut ecosystems [181]. Comparable findings were observed in models targeting inflammation-associated pathobionts, in which lytic phage cocktails against *Klebsiella pneumoniae* reduced intestinal and hepatobiliary inflammation in IBD and primary sclerosing cholangitis, while causing minimal disruption to commensal microbial communities [182,183]. Together, these findings support the concept of phage therapy as a microbiome-modulating strategy that combines pathogen-specific targeting with preservation of microbial homeostasis.

At the same time, the growing interest in phage therapy reflects the urgent need for alternatives to conventional antibiotics against MDR pathogens, particularly in chronic and biofilm-associated infections. Experimental and early clinical studies involving *Pseudomonas aeruginosa* demonstrated encouraging therapeutic effects, but also highlighted important limitations, including incomplete pathogen eradication, biofilm persistence, and rapid emergence of phage-resistant mutants [184,185].

These observations suggest that phage therapy may be most effective when integrated with complementary strategies. Indeed, phage–antibiotic combinations improved clearance of MDR *Salmonella*, delayed resistance emergence, reduced biofilm persistence, and better preserved gut microbial diversity compared with antibiotic treatment alone [186]. In parallel, increasing attention has focused on optimized phage cocktail design, including computational and personalized approaches intended to broaden host coverage and reduce selection of resistant bacterial mutants [187,188]. Similar findings in *Enterococcus* and *Klebsiella* models further support adaptive multi-phage formulations as a promising strategy for precision microbiome modulation [188,189].

Recent studies have also shown that phage cocktails can selectively eliminate antibiotic-resistant pathogens such as *C. difficile* while favoring the expansion of beneficial bacterial populations [178]. In addition to direct antimicrobial activity, phages may influence host metabolism and immune responses, opening new perspectives for microbiome-targeted interventions in disorders associated with gut dysbiosis, including obesity and cancer [174]. Despite these encouraging findings, current evidence remains largely preclinical or limited to early clinical investigations. Translation into routine practice is constrained by the narrow host range of many phages, rapid emergence of phage-resistant bacterial mutants, manufacturing and quality-control challenges, regulatory uncertainty, and the need for personalized phage selection and standardized susceptibility testing. Consequently, phage therapy should currently be regarded as a promising but still investigational microbiome-targeted strategy whose broader clinical implementation will require robust clinical trials and harmonized production and regulatory frameworks.

### 6.3. FMT

FMT is another strategy for gut microbiota restoration, particularly in recurrent *Clostridioides difficile* infection (CDI). Conventional donor-derived FMT involves the transfer of fecal microbiota from a screened healthy donor to a recipient with dysbiosis, aiming to restore microbial diversity, colonization resistance, and metabolic function [177,190]. However, the regulatory landscape has changed substantially with the approval of standardized microbiota-based products. Rebyota (fecal microbiota, live-jslm) was approved by the FDA in 2022 for the prevention of recurrent CDI in adults following antibacterial treatment, and is administered rectally as a single-dose microbiota suspension [191]. Vowst/SER-109 (fecal microbiota spores, live-brpk) was approved in 2023 as the first orally administered fecal microbiota product for the prevention of recurrent CDI in adults after antibacterial treatment [192]. These products should therefore be distinguished from conventional FMT because they are manufactured, quality-controlled microbiota-based therapeutics rather than individually prepared donor stool preparations.

FMT and microbiota-based therapeutics have demonstrated their clearest clinical utility in recurrent CDI, where antibiotic-induced microbiota depletion facilitates *C. difficile* overgrowth. By restoring colonization resistance and microbial diversity, they suppress pathogen expansion and support immune and metabolic homeostasis [14]. The clinical evidence for oral SER-109/Vowst is supported by the phase 3 ECOSPOR III trial, in which purified *Bacillota* spores were superior to placebo in reducing CDI recurrence after standard-of-care antibiotics [193]. Current IDSA/SHEA guidance recognizes microbiota restoration as an option for patients with multiple CDI recurrences after appropriate antibiotic therapy, while emphasizing careful patient selection and safety considerations [194]. Beyond CDI, applications in IBD, irritable bowel syndrome, obesity-related conditions, metabolic dysfunction, and MDR organism decolonization remain investigational and should not be interpreted as established indications [174].

Recent studies indicate that FMT efficacy depends not only on microbial transfer itself, but also on donor-derived strain engraftment, ecological compatibility, and long-term microbiome stabilization. Successful donor strain engraftment has been associated with improved clinical outcomes across recurrent CDI, ulcerative colitis, and metabolic disorders, whereas non-responders frequently exhibit limited persistence of donor-derived taxa and reduced ecological integration [195,196,197]. Strain-level analyses further demonstrated that recipient baseline dysbiosis, donor microbial composition, pretreatment strategies, and microbiome diversity substantially influence engraftment success and post-FMT resilience [195,198]. Emerging ecological models suggest that post-FMT recovery involves complex processes of community coalescence, strain displacement, and ecological stabilization rather than complete replacement of the donor microbiota [199].

Growing evidence supports the transition from empiric FMT toward more personalized and function-oriented microbiome therapeutics. In ulcerative colitis, enrichment of *Lachnospiraceae* and *Ruminococcaceae* has been associated with favorable therapeutic responses, whereas *Prevotellaceae*-dominant profiles correlated with treatment failure [200]. Similarly, donor-dependent variability in microbial engraftment and therapeutic durability has been observed in studies evaluating FMT combined with dietary modulation strategies [201]. In irritable bowel syndrome, both encapsulated and rectally administered FMT improved symptom severity and quality of life compared with placebo, supporting the feasibility of less invasive oral delivery approaches [202]. Encapsulated lyophilized FMT has also shown potential for partial decolonization of ESBL-producing and carbapenem-resistant *Enterobacterales*, further highlighting the emerging role of microbiota-targeted interventions in AMR management [203]. However, these uses remain investigational and require stronger controlled clinical validation.

Safety remains a central limitation of conventional FMT. FDA safety communications have reported serious infections, including transmission of multidrug-resistant organisms, following investigational FMT, leading to strengthened donor-screening requirements for MDR organisms and other transmissible pathogens [204]. Because donor-derived material contains complex live microbial communities, rigorous donor selection, standardized pathogen screening, traceability, manufacturing controls, and post-administration monitoring are essential. The emergence of approved products such as Rebyota and Vowst partly addresses these issues through regulated manufacturing and quality-control pathways, but they do not eliminate the need for continued pharmacovigilance and careful risk assessment, particularly in immunocompromised or medically complex patients. Consequently, the field is moving from empiric donor-derived FMT toward standardized, indication-specific microbiota-based therapeutics designed to improve safety, reproducibility, and clinical efficacy.

### 6.4. Other Non-Conventional Strategies

Beyond probiotics, bacteriophages, and fecal microbiota transplantation, several emerging approaches aim to restore microbial ecosystem function with greater precision. One promising strategy involves the use of defined bacterial consortia, in which carefully selected bacterial strains are combined to re-establish microbial diversity and colonization resistance while avoiding the variability and safety concerns associated with conventional FMT. Unlike donor-derived fecal material, these standardized microbial communities enable controlled composition, improved manufacturing reproducibility, and reduced risk of pathogen transmission. Early clinical studies have demonstrated encouraging results for recurrent *Clostridioides difficile* infection and support further the development of next-generation live biotherapeutic products for microbiome restoration [177,205].

Bacteriocins represent another emerging microbiome-targeted strategy. These ribosomally synthesized antimicrobial peptides, naturally produced by commensal bacteria, selectively inhibit pathogenic microorganisms while largely preserving beneficial microbial communities. In addition to their antimicrobial activity, bacteriocins exhibit immunomodulatory properties and may promote restoration of gut homeostasis with less collateral disruption than conventional antibiotics. Although current evidence remains predominantly preclinical, their selective mechanism of action makes them attractive candidates for future microbiota-directed therapies targeting antibiotic-associated dysbiosis and antimicrobial resistance [16,206,207].

Overall, these emerging approaches seek to restore microbiome function while minimizing the ecological disruption associated with broad-spectrum antibiotics. Although further clinical validation is required, they illustrate the ongoing transition from nonspecific microbial replacement strategies toward precision microbiome engineering.

## 7. Conclusions and Future Perspectives

The gut microbiota is a highly dynamic, functionally integrated ecosystem whose resilience is profoundly affected by antibiotic exposure. Although indispensable for the treatment of bacterial infections, antibiotics can induce persistent reductions in microbial diversity, depletion of beneficial commensals, disruption of colonization resistance, metabolic dysregulation, and expansion of antimicrobial-resistant pathobionts, with consequences extending far beyond the gastrointestinal tract [208]. Increasing evidence suggests that antibiotic-induced dysbiosis may contribute to broader immune and metabolic disturbances, although the strength of the evidence varies across clinical conditions. The clearest evidence currently supports its role in antibiotic-associated diarrhea and *C. difficile* infection, whereas links with chronic inflammatory, metabolic, autoimmune, and neuropsychiatric disorders remain under active investigation.

By integrating ecological disturbances, resistome dynamics, metabolic consequences, and microbiome restoration strategies within a single framework, this review provides an updated perspective that extends beyond descriptive taxonomic changes and emphasizes the importance of microbiome resilience and functional recovery following antibiotic exposure. Nonetheless, several limitations of this paper should be acknowledged. As a narrative review, the present work is inherently subject to selection bias and does not follow a formal systematic review methodology. Moreover, substantial heterogeneity among currently available studies, including differences in study design, antibiotic regimens, sequencing platforms, analytical pipelines, and population characteristics, complicates direct comparisons and limits the generalizability of the findings. Technical factors such as batch effects, DNA extraction methods, primer selection, sample collection and storage conditions, sequencing biases, and limitations of current taxonomic reference databases further contribute to variability across studies. In addition, many mechanistic insights originate from preclinical models and require validation in well-designed longitudinal human studies.

Despite considerable progress, several important questions remain unresolved. Future research should prioritize: (i) elucidating the mechanisms governing long-term resistome persistence and horizontal gene transfer following antibiotic exposure; (ii) identifying reliable microbial and functional biomarkers of microbiome resilience and recovery; (iii) establishing standardized approaches for evaluating microbiome-targeted interventions; (iv) defining the contribution of microbial metabolites to systemic immune and metabolic disturbances; and (v) developing personalized microbiome restoration strategies based on host and microbial characteristics.

Advances in metagenomics, metabolomics, transcriptomics, single-cell sequencing, and artificial intelligence-driven ecological modeling are expected to facilitate identification of microbial and functional signatures associated with antibiotic-induced dysbiosis, treatment responsiveness, and disease susceptibility [174,177]. These technologies may enable the development of personalized microbiome-based diagnostics and targeted therapeutics tailored to host genetics, age, diet, immune status, and environmental exposures. In particular, integrating bioinformatics tools with multi-omics approaches may improve the prediction of donor–recipient compatibility, microbial engraftment, and long-term ecological stability following FMT and other microbiome-based therapies [195,198,199]. In parallel, synthetic biology approaches, including genetically engineered probiotics and designer microbial consortia, may provide next-generation strategies for restoring microbial resilience and enhancing host resistance to dysbiosis-associated diseases [174,178].

Early-life microbiota establishment represents an important determinant of long-term microbiome resilience and immune maturation. Consequently, minimizing unnecessary antibiotic exposure during pregnancy and early childhood may help preserve microbial development during this vulnerable period [174,177].

From a clinical perspective, preserving microbiome homeostasis should accompany antimicrobial stewardship efforts. Judicious antibiotic prescribing, avoidance of unnecessary broad-spectrum therapies, optimization of treatment duration, and the rational implementation of evidence-based microbiome-targeted interventions may become essential for reducing the long-term health burden associated with antibiotic-induced dysbiosis and AMR [25].

Ultimately, maintaining microbial ecosystem integrity should be considered an integral component of precision microbiome medicine and antimicrobial stewardship, supporting both long-term host health and efforts to limit the emergence and dissemination of antimicrobial resistance.

## Figures and Tables

**Table 1:** Emerging concepts redefining healthy gut microbiome architecture and functionality.

Concept/Approach	Main Findings	Relevance for Healthy Microbiome Definition	References
Functional redundancy	Phylogenetically distinct microorganisms can perform overlapping metabolic functions, preserving ecosystem performance despite taxonomic variation.	Supports functional stability and resilience following ecological perturbations	[42]
Metabolism-centric microbiome assessment	Microbiome health is increasingly defined by metabolic activity rather than taxonomic composition alone.	Promotes function-oriented evaluation of microbiome status.	[44]
Healthy microbiome as a context-dependent state	No universal healthy microbiome exists; composition and function are shaped by host genetics, diet, lifestyle, age, and environmental factors.	Emphasizes individualized definitions of microbiome health.	[45]
Microbiome resilience	Healthy microbial communities maintain functional stability and recover following dietary or environmental disturbances.	Reflects adaptive capacity and restoration potential after perturbations.	[46]
Host–microbiome–environment interactions	Gut physiology, intestinal transit, luminal pH, diet, and environmental exposures collectively influence microbial composition and metabolic outputs.	Highlights the importance of host context in maintaining microbiome function.	[43]
Keystone taxa ecology	Low-abundance microorganisms may exert disproportionate effects on community organization, metabolic interactions, and ecosystem stability.	Identifies critical taxa supporting microbiome resilience and homeostasis.	[59]
Systems-level microbiome characterization	Integration of metagenomics, metaproteomics, metabolomics, and ecological modeling enables functional characterization beyond taxonomy.	Supports comprehensive assessment of microbiome functionality and resilience	[41]

**Table 2:** Early-life factors influencing vulnerability to antibiotic-associated dysbiosis and resistome expansion.

Factor	Main Microbiota Changes	Potential Long-Term Outcomes	References
Vaginal delivery	Promotes maternal microbial transmission and enrichment of *Bifidobacterium* and *Bacteroides*	Enhances colonization resistance and microbiome resilience	[72,73]
Cesarean delivery	Delayed anaerobic colonization; increased ARG burden	Greater susceptibility to dysbiosis and impaired microbiome recovery	[73,91]
Breastfeeding	Supports *Bifidobacterium* enrichment and lower ARG burden	Improves microbiome stability and limits resistome expansion	[74,75]
Formula feeding	Reduced beneficial anaerobes; altered microbial metabolism	Associated with higher ARG carriage and reduced microbial resilience	[92]
Antibiotic exposure	Loss of microbial diversity, depletion of commensals, transient ARG expansion	Major driver of antibiotic-associated dysbiosis and delayed microbiome recovery	[76,93]
Prematurity/NICU exposure	Immature microbiome; frequent antibiotic exposure; opportunistic taxa expansion	Increased risk of persistent dysbiosis, MDR colonization, NEC, and LOS	[93,94]

**Table 3:** Major antibiotic-associated alterations of gut microbiota and related functional consequences.

Antibiotic Class	Primary Ecological Effect	Key Depleted/Enriched Taxa	Major Functional Consequences	Resistome/ARG Impact	References
Broad-spectrum β-lactams	Reduced diversity; depletion of obligate anaerobes	↓ Bacteroides, SCFA producers	↓ SCFAs, impaired barrier integrity	Selection of β-lactam ARGs; dysbiosis-associated resistome expansion	[129,132]
Carbapenems	Severe depletion of anaerobic commensals	↓ Colonization-resistant taxa	Metabolic dysfunction; impaired colonization resistance	Expansion of carbapenem-resistant Enterobacterales	[129,130,131,132]
Macrolides	Delayed ecological recovery	Persistent compositional shifts	Reduced ecological resilience	Long-term resistome remodeling	[124]
Glycopeptides	Loss of Gram-positive commensals	↓ Bacillota; ↓ SCFA producers	↓ Acetate; impaired IL-17-mediated antifungal immunity	Selection of glycopeptide resistance determinants	[127,131]
Fluoroquinolones	Persistent loss of diversity	Enrichment of resistant Enterobacterales	Long-lasting ecological disruption	Fluoroquinolone ARG enrichment	[125]
Broad-spectrum antibiotic cocktails	Collapse of keystone taxa	↓ *B. bifidum*, *A. muciniphila*	Metabolic dysfunction, immune dysregulation, delayed recovery	Multi-class ARG enrichment	[122,126,128,134]

Reported microbiota alterations may vary substantially across studies depending on antibiotic exposure regimen, host-related factors, sequencing methodologies, and sampling time points; The downward arrow (↓) indicates a reduction or depletion in the abundance of the indicated taxa or metabolite compared with baseline.

**Table 4:** Comparison of current microbiota restoration strategies after antibiotic-induced dysbiosis. Created based on information from [156,157,158,159].

Strategy	Major Strengths	Current Limitations	Main Clinical Applications
Probiotics	Safe, accessible, immune modulation	Strain-dependent efficacy; variable engraftment	AAD prevention, CDI adjunct, IBD
Prebiotics	Promote beneficial taxa and SCFA production	Limited efficacy in severe dysbiosis	Metabolic support, inflammation
Postbiotics	High stability; no live microorganisms	Limited standardization and clinical evidence	Gut barrier support, inflammation
FMT	Most comprehensive microbiota restoration	Donor screening; safety and standardization issues	Recurrent CDI; investigational use in IBD and MDR decolonization
Bacteriophages	High pathogen specificity; microbiota preservation	Limited clinical evidence; resistance development	MDR infections; severe dysbiosis
Bacteriocins	Selective antimicrobial activity; immunomodulation	Mostly preclinical evidence	Experimental microbiota restoration

FMT, fecal microbiota transplantation; AAD, antibiotic-associated diarrhea; CDI, *Clostridioides difficile* infection; IBD, inflammatory bowel disease; MDR, multidrug resistant.

## Data Availability

No new data were created or analyzed in this study. Data sharing is not applicable to this article.

## Abbreviations

The following abbreviations are used in this manuscript:

ARGs	Antibiotic resistance genes
AAD	Antibiotic-associated diarrhea
AMR	Antimicrobial resistance
SCFAs	Short-chain fatty acids
MDR	Multidrug-resistant
MGEs	Mobile genetic elements
FMT	Fecal microbiota transplantation
LOS	Late-onset sepsis
NEC	Necrotizing enterocolitis
NICU	Neonatal intensive care unit
CDI	*Clostridioides difficile* infection
CRE	Carbapenem-resistant Enterobacteriaceae
HGT	Horizontal gene transfer
ESBL	Extended-spectrum beta-lactamase
IBD	Inflammatory bowel disease
